# Combinatorial selection of molecular conformations and supramolecular synthons in quercetin cocrystal landscapes: a route to ternary solids

**DOI:** 10.1107/S2052252515009884

**Published:** 2015-06-11

**Authors:** Ritesh Dubey, Gautam R. Desiraju

**Affiliations:** a Solid State and Structural Chemistry Unit, Indian Institute of Science, Bangalore 560 012, India

**Keywords:** supramolecular synthons, polymorphism, quercetin, cocrystals, crystal structure landscape

## Abstract

The crystallization of 28 binary and ternary cocrystals of quercetin with dibasic coformers is analyzed in terms of a combinatorial selection from a solution of preferred molecular conformations and supramolecular synthons. The crystal structures are characterized by distinctive O—H⋯N and O—H⋯O based synthons and are classified as nonporous, porous and helical. Variability in molecular conformation and synthon structure led to an increase in the energetic and structural space around the crystallization event. This space is the crystal structure landscape of the compound and is explored by fine-tuning the experimental conditions of crystallization. In the landscape context, we develop a strategy for the isolation of ternary cocrystals with the use of auxiliary template molecules to reduce the molecular and supramolecular ‘confusion’ that is inherent in a molecule like quercetin. The absence of concomitant polymorphism in this study highlights the selectivity in conformation and synthon choice from the virtual combinatorial library in solution.

## Abstract 2

Crucial ‘purification’ steps inherent to the ‘selection’ of particular molecular conformations and supramolecular synthons in solution leads to a successful synthetic strategy for ternary solids, a normally difficult exercise in crystal engineering. This work also suggests a parallel between such strategies and combinatorial chemistry.

## 1. Introduction   

Polymorphism (Groth, 1906–1919 ▸; Deffet, 1942 ▸) is intrinsic to organic compounds and multiple crystalline phases exist because of contrasting kinetic and thermodynamic preferences during crystallization (Desiraju, 2002 ▸, 2007 ▸, 2013 ▸). The phenomenon may be more common for molecules which have both conformational flexibility and hydrogen-bonding propensity, although it is by no means unknown to other categories of substances (Sarma & Desiraju, 1999 ▸; Desiraju, 1997*b* ▸). Conformationally flexible molecules provide alternative packing arrangements because rotations around single bonds afford differently shaped molecules (Nangia, 2008 ▸; Amadei *et al.*, 1998 ▸; Bhatt & Desiraju, 2007 ▸; Bond *et al.*, 2007*a* ▸,*b* ▸). Again, the presence of multiple hydrogen bond functionalities facilitates the emergence of several crystalline forms because of the different supramolecular synthon possibilities that could ensue (Jetti *et al.*, 2003 ▸; Roy & Matzger, 2009 ▸; Chen *et al.*, 2005 ▸; López-Mejías *et al.*, 2012 ▸). The (molecular) energy differences between conformations is comparable to the (crystal) energy differences between polymorphs (<20 kJ mol^−1^), and therefore a combination of both factors enhances the likelihood of polymorphism in organic compounds, especially if small changes in experimental conditions result (Nangia, 2008 ▸). This energy profile may contain various dynamically equilibrated molecular conformations and supramolecular synthons in solution that, in combination, would be capable of providing a large number of crystalline phases from the supersaturated crystallizing medium. These phases, which would be virtual save for some extenuating circumstance, may be amplified by solvent control or by using suitable template molecules to give isolable solids (Blagden & Davey, 2003 ▸; Kulkarni *et al.*, 2012 ▸; Staab *et al.*, 1990 ▸; Friščič & MacGillivray, 2009 ▸). If we consider the crystal as a supramolecular entity (Desiraju, 1996 ▸), both factors, molecular conformation and multiplicity of synthons, collectively increase the energetic and structural space around the reaction trajectories in this supramolecular synthesis. This space is the *crystal structure landscape* of the compound (Thakur *et al.*, 2015 ▸; Dubey *et al.*, 2012 ▸, Dubey & Desiraju, 2014*a* ▸,*b* ▸).

The concept of polymorphism is very well studied in single component systems but systematic exploration of cocrystal polymorphism has only recently begun in the crystal engineering context (Aitipamula *et al.*, 2014 ▸). We have performed a detailed landscape exploration based on the conformationally flexible orcinol (5-methylresorcinol) molecule and its cocrystals with N-bases (Mukherjee *et al.*, 2011 ▸). We found that all possible conformations of the orcinol molecule can exist in these crystalline forms: *syn*–*syn* (four cocrystals), *anti*–*anti* (seven cocrystals), *syn*–*anti* (five cocrystals). We further showed that the orcinol:4,4′-bipyridine (ORC:44BP) cocrystal landscape consists of five anhydrous crystal forms, but that all forms have just one of the orcinol conformations (*anti*–*anti*), however, with different packing arrangements (Dubey *et al.*, 2014 ▸). Cocrystal polymorphism can also involve changes in the main synthons themselves (Desiraju, 1995 ▸), the so-called synthon polymorphism (Sreekanth *et al.*, 2007 ▸; Mukherjee & Desiraju, 2011 ▸). The 4-hydroxybenzoic acid:4,4′-bipyridine cocrystal, for example, shows efficient synthon selection with different experimental conditions leading to fundamentally different crystal forms (Mukherjee & Desiraju, 2011 ▸). Synthon polymorphism in cocrystals may be considered to be combinatorial selection from a virtual synthon library. This idea of selection has been elaborated in solution chemistry previously (Lehn, 1999 ▸, 2007 ▸). Recently, we interpreted the extensive polymorphic modifications in phloroglucinol:1,2-bis(4-pyridyl)ethylene (PGL:DPE) and phloroglucinol:phenazine (PGL:PHE) cocrystal systems on this basis and showed selective amplification of synthons using auxiliary template molecules (Dubey & Desiraju, 2014*a* ▸).

Quercetin (QUE) is an important polyphenol flavonoid which shows a variety of biological properties including antioxidant behavior (Grassi *et al.*, 2010 ▸; Zhang *et al.*, 2011 ▸). Using the cocrystal landscapes of quercetin (Kavuru *et al.*, 2010 ▸) with dibasic coformers, *i.e.* tetramethylpyrazine (TMP), 4,4′-bipyridine (44BP), 1,2-bis(4-pyridyl)ethylene (DPE-1), 1,2-bis(4-pyridyl)ethane (DPE-2), 4,4′-azopyridine (44AP), phenazine (PHE), we extend here the idea of a combinatorial library towards prenucleation events where a conformationally flexible compound has a large number of virtual conformations. This approach allows one to consider the aggregation of these flexible molecules towards heteroclusters as synthon selection from a virtual library leading eventually to nucleation events and the final crystal structure (Desiraju, 1997 ▸ *a* ▸; Davey *et al.*, 2013 ▸).

## 2. Experimental   

All datasets were collected on a Rigaku Mercury 375R/MCCD (XtaLABmini) diffractometer with a graphite monochromator using Mo *K*α radiation, attached with a Rigaku low-temperature gas spray cooler. The data were processed with *CrystalClear* software (Rigaku, 2009 ▸). QUE:DPE-II (form III) was collected on an Oxford Xcalibur diffractometer with a microfocus X-ray source (Mo *K*α) equipped with a Cryojet-HT nitrogen gas stream cooling device and data were processed with *CrysAlisPro* (Oxford, 2009 ▸). Data for some of the crystals, the QUE:DPE-I (form IV), QUE:DPE-II (form II), QUE:44AP (form II), QUE:PHE (forms III and IV) and QUE:DPE-I:ANT were collected on a Bruker Kappa Apex II CCD diffractometer using Mo *K*α radiation and an Oxford cryosystems N_2_ open-flow cryostat (Bruker, 2006 ▸). Cell refinement, data integration and data reduction were carried out using the *SAINT-Plus* program. Crystal structures were solved by direct methods and refined in the spherical-atom approximation using *SHELXL*2012 (Sheldrick, 2008 ▸) from the *WinGX* suite (Farrugia, 1999 ▸). All non-H atoms were refined anisotropically. The H atoms were fixed on a riding model and acidic H atoms were located *via* Fourier maps. Precise experimental details of each crystal structure are provided in the supporting information.

## 3. Results and discussion   

This section is divided into a description of the landscapes and the combinatorial analysis of the crystallization events. The primary driving force in the formation of a cocrystal is enthalpically driven synthon formation in solution that involves two or more chemically distinct molecules. A particular synthon is also associated with certain conformations of the QUE molecule. Which conformation and which synthon is preferred in any instance depend on the structure of the coformer, the presence of auxiliary molecules and other experimental variables.

### 3.1. Quercetin cocrystal landscapes   

**Figure 1:**
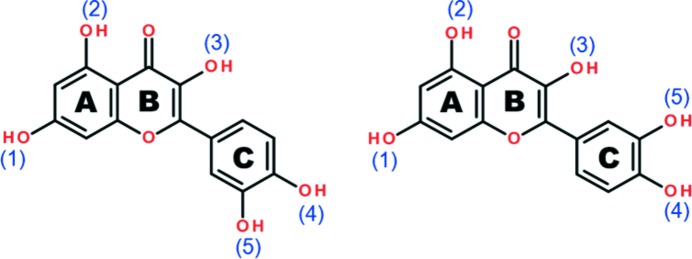
Flipping of the (5) OH group by rotation of the flexible C—C single bond in quercetin.

**Figure 2:**
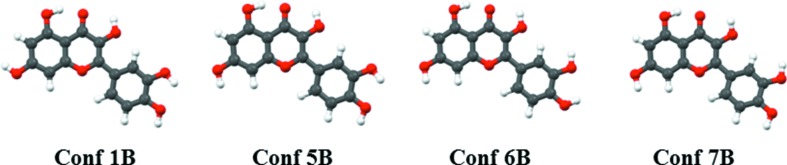
Molecular conformations in quercetin cocrystal landscapes.

Quercetin has five hydroxy groups (Fig. 1 ▸) and the mutual orientations of these groups provide a variety of putative conformations that constitute a virtual conformational library (supporting information). This scenario is further complicated by the conformational possibilities brought about with the flexible C-ring. We performed extensive cocrystallization experiments with dibasic coformers (supporting information). These crystal structures are characterized by several O—H⋯N and/or O—H⋯O based supramolecular synthons which are all rather distinctive and lead to structure types that we refer to in this paper as *nonporous*, *porous* and *helical* within the extended domain of the quercetin cocrystal landscapes. There are other synthon possibilities which were revealed in later cocrystal screening experiments, giving a hint about the virtual nature of this library. Figs. 2 ▸ and 3 ▸ illustrate quercetin conformations and a schematic representation of the supramolecular synthons discussed here. In general, the second (OH2) and third (OH3) hydroxy groups of quercetin are conformationally locked by intramolecular hydrogen bonding, while OH1, OH4 and OH5 are responsible for the supramolecular development of various intricate hydrogen bonding patterns in the crystal structures. The supporting information gives a list of 12 conformations and their energies. It is interesting that the most stable conformation (*Conf 2B*) does not feature in the experimental crystal structures. This could be because of a trade-off between intra- and intermolecular factors.

In the quercetin–tetramethyl­pyrazine (QUE:TMP) cocrystal system, we were able to isolate four crystal forms: two anhydrates, a 1,4-dioxane solvate and a tetrahydrofuran (thf) solvate. The crystal stoichiometries can be 1:3, 1:2 or 1:1. QUE is underexpressed in the crystal in relation to the amount taken in solution for crystallization. So a 1:3 anhydrate, form I (space group *Pc*), is obtained from a solution that contains QUE and TMP in a 1:1 ratio, while a 1:2 anhydrate, form II (space group *P*2_1_/*c*), is obtained when the QUE:TMP ratio taken in solution is 2:1. In form I, quercetin acquires the *Conf 6B* conformation that accelerates the selection of *synthon A* from the solution library. In this crystal structure, OH1, OH2, OH3 and OH4 groups are involved in O–H⋯N hydrogen bonding while OH2 and OH5 groups participate with O–H⋯O hydrogen bonds. Form II also has *Conf 6B*, but a different supramolecular synthon, *synthon B*. So we observe a preferential trend of O—H⋯O over O—H⋯N with increasing QUE content in solution, as might be expected. This trend continues when the QUE content is increased further. When QUE and TMP are taken in a 3:1 ratio in solution, the outcome are two 1:1 cocrystals, form III and form IV, both of which have *Conf 5B* and *synthon E*. In these crystal structures, the molecular conformation and synthon selection mutually provide a channel arrangement that facilitates porosity along [100]. This one-dimensional porous channel is filled with dioxane and thf solvents as a guest, respectively. In the crystal structure landscape context this movement from a close packed to a porous structure highlights that subtle factors underlie the selection of certain high-energy conformations leading to particular synthons and hydrogen bond arrangements. This combinatorial selection of molecular conformation as well as the amplification of ‘correct’ synthons could be delicately controlled by changing the experimental conditions – stoichiometric ratios or solvents – during crystallization.

**Figure 4:**
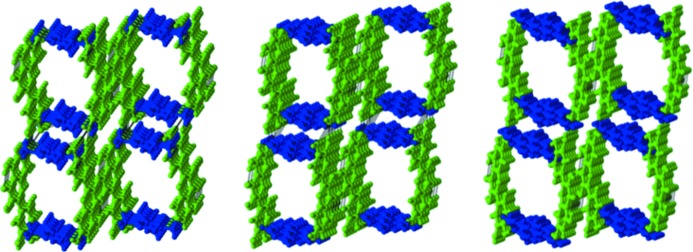
Porous molecular arrangements in quercetin cocrystal landscapes; QUE:TMP (left), QUE:44BP (middle), QUE:DPE (right). Compounds are color coded: green – quercetin; blue – coformer.

The quercetin–4,4′-bipyridine cocrystal system (QUE:44BP) has four crystal forms. Three of them are porous, with basically the same structure as the porous QUE:TMP crystals, and have 1:1 stoichiometry. As in QUE:TMP, these forms have the same conformation *Conf 5B* and the same *synthon E*. These crystal structures accommodate 1,4-dioxane, thf and even coformer (44BP) as a guest in the larger porous pocket (Fig. 4 ▸). When QUE and 44BP were taken in 1:4 ratios in solution for crystallization, a fourth form was obtained in which QUE switches its molecular conformation to the high energy *Conf 7B*. This crystal form is a 1:3 QUE:44BP monohydrate with masked *synthon F* where water is involved in complex hydrogen bond patterns. MacGillivray *et al.* have utilized the appropriate term *masked synthon* to describe a situation wherein water actively participates in the development of the supramolecular synthon (Sander *et al.*, 2013 ▸). In the next step, we replaced the 44BP coformer with 1,2-bis(4-pyridyl)ethylene (DPE-I) and explored its corresponding structural space. The QUE:DPE-I landscape contains five crystal forms – two anhydrates and one solvate each of 1,4-dioxane, thf and DMF. The latter pseudopolymorphic structures are all porous with the solvents situated in the open pockets. Extending the argument, we have described recently that replacement of DPE-I with 1,2-bis(4-pyridyl)ethane, DPE-II, can extend the structural landscape (Dubey & Desiraju, 2015 ▸). In the present study we made cocrystals of QUE with DPE-II and also with 4,4′-azopyridine (44AP), which is chemically similar to DPE-I and DPE-II. We found the same conformation *Conf 6B* and porous *synthon E* in these structures.

**Figure 5:**
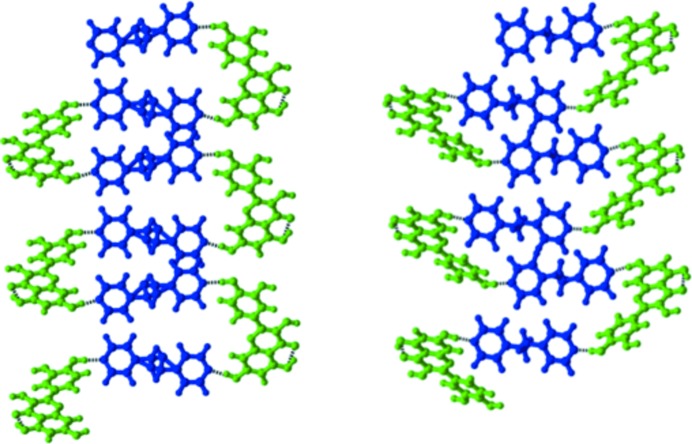
Helical molecular arrangements in quercetin cocrystal landscapes. Compounds are color coded: green – quercetin; blue – coformer.

Let us now consider the anhydrates. By altering the experimental conditions, we found that crystallization of a 1:1 ratio of QUE and DPE-I provides two forms: the first has a 1:1 QUE:DPE-I stoichiometry with *Conf 1B* and *synthon H*, while the other is a 1:2 QUE:DPE-I cocrystal with *Conf 7B* and *synthon G*. The latter illustrates a new helical structure type illustrated in Fig. 5 ▸, which shows different molecular and supramolecular features from the previously described nonporous and porous structure types and correspondingly enhances the domain of the structural space available. Similar to the pseudopolymorphs, the anhydrates also follow the same trend after coformer replacement of DPE-I by DPE-II or 44AP and hints about the robustness of conformation and synthon selection during crystallization. Changing the experimental conditions for QUE:TMP, QUE:44BP and QUE:DPE-I allows one to move within a landscape from nonporous to porous and thereafter to helical crystal structures. In most cases the corresponding DPE-I, DPE-II and 44AP cocrystal structures are isomorphous. However, in the 2:1 QUE:DPE-II cocrystal there is a different *synthon C* despite the same conformation *Conf 6B*. There are a number of structures lying within a small energy window, all with the same conformation but different synthon possibilities. We did not observe any instance of concomitant polymorphism so selectivity in synthon choice is clearly present. The crystallographic details of the crystal structures are given in the supporting information.

**Figure 6:**
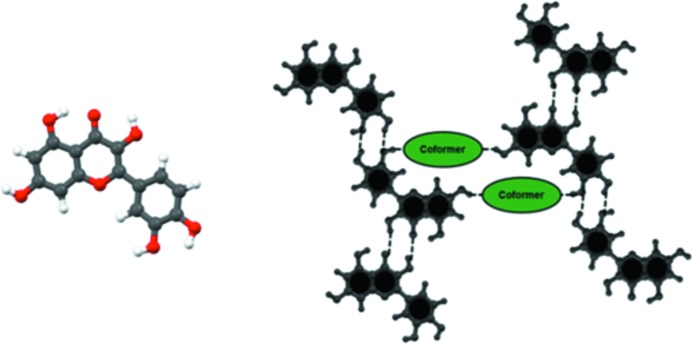
Selection of molecular conformation, *Conf 2A*, and supramolecular synthon, *synthon I*, in the anhydrous 2:3 QUE:PHE crystal structure.

Finally, the quercetin–phenazine cocrystal landscape (QUE:PHE) is very comparable and has five crystal forms: two anhydrates, a methanolate, a 1,4-dioxane solvate and a monohydrate. Of these crystal forms, two are porous with *Conf 5B* and *synthon E* and their one-directional porous channel is occupied by 1,4-dioxane and PHE as a guest molecule. In the methanolate, *Conf 6B* is taken with *synthon D*. PHE is geometrically different from DPE-I and DPE-II, but it still amplifies *Conf 7B* in the monohydrate; this conformation is favored by DPE-I and DPE-II in their helical structures. However, in the case of the QUE–PHE landscape the rigid nature of the small coformer molecule PHE prevents adoption of the helical structure for the monohydrate. The observed structure is nonporous with an alternative supramolecular synthon, *synthon G*. The final structure is the second anhydrate where the hitherto virtual conformation *Conf 2A* is expressed as also *synthon I* that had not been observed previously (Fig. 6 ▸). These unique selections of conformation and supramolecular synthons highlight the virtuality of conformational and synthon libraries in supersaturated solution that could selectively be fine-tuned by experimental conditions. So, in general, the QUE:PHE cocrystal landscape shows the same conformations selection: *Conf 6B*, *Conf 5B* and *Conf 7B* as in the previous landscapes. These conformations lead respectively to each of the three structure types: nonporous, porous and helical.

In summary, deliberate coformer replacement is a chemical probe that may be used to perturb and understand kinetic events during crystallization that are otherwise experimentally inaccessible. The coformers TMP, 44BP, DPE-I and PHE are chemically similar but contain different geometrical features and topologies. The chemical similarity could be the factor that leads to a common conformation. The geometrical dissimilarities could be the factors that lead to a difference in stabilities between the structure types, in this case nonporous, porous and helical.^1^ We note that this experimental perturbation would help one to understand the crystallization kinetics and accordingly would enter into more remote domains in the molecule to crystal landscape pathway.

### 3.2. Auxiliary template molecules and ternary cocrystals   

This detailed understanding of quercetin cocrystal landscapes can lead one towards the isolation of stoichiometric ternary molecular solids. Very few reports discuss the synthesis of cocrystals that contain three molecules that all exist as solids at room temperature in their native crystal structures. There are only a handful of design strategies available (Aakeröy *et al.*, 2000 ▸; Bhogala & Nangia, 2008 ▸; Thothadi & Desiraju, 2013 ▸; Seaton *et al.*, 2013 ▸; Chakraborty *et al.*, 2014 ▸; Dobrowolski *et al.*, 2014 ▸). This exercise requires a good control over intermolecular interactions because there is an inherent tendency during crystallization to exclude ‘impurities’. In the context of quercetin cocrystal landscapes, any design strategy for ternary solids should be based on a precise selection of molecular conformations and supramolecular synthons during molecular recognition leading eventually to convergence into a modular and robust crystal structure (Sanders, 2004 ▸; Cougnon & Sanders, 2011 ▸). We utilized our understanding of the events as outlined in the earlier section and used appropriate auxiliary template molecules to reduce the molecular and supramolecular ‘confusion’ that is inherent in a molecule like quercetin.

**Figure 7:**
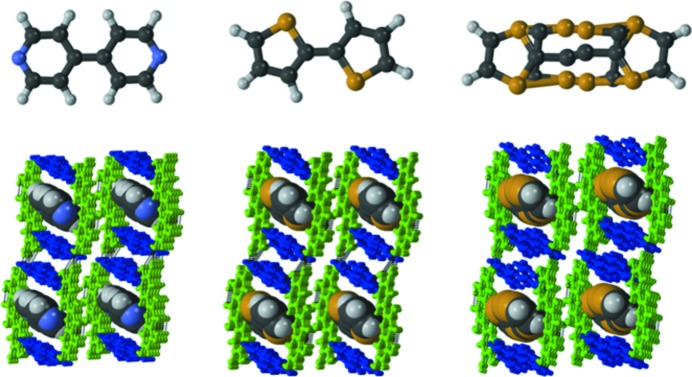
Ternary design strategy. 22TP as a template molecule in QUE:44BP and QUE:DPE-I cocrystals. The presence of the robust *Conf 5B* conformation and supramolecular synthon, *synthon E*, is presented in the ternary solids. Respective guest molecules are shown.

**Figure 8:**
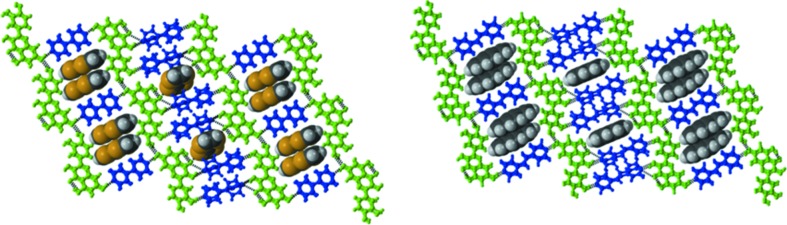
Ternary design strategy. TTF (top) and PYR (lower) as template molecules in QUE:44BP and QUE:DPE-I cocrystals. These crystal structures also highlight tghe virtual selection of *Conf 6B* and *synthon B*.

The design strategy for ternary solids depends upon the QUE:44BP landscape in which there are consistent appearances of *Conf 5B* and *synthon E* from the pool of possibilities. In our ternary design we exploited these robust chemical features and a 1:1 solution of QUE and 44BP in ^*i*^PrOH solution was layered with a saturated toluene solution of 2,2′-bis-thiophene (22TP) so that liquid diffusion takes place. The appearance of a 2:2:1 stoichiometric ternary solid with *Conf 5B* conformation and *synthon E* validated our anticipation and hints at the utility of the landscape idea in designing complex supramolecular architectures. We have shown shape and size mimicry of 22TP for 44BP in ternary solids earlier (Tothadi *et al.*, 2011 ▸). Using this molecular mimicry as a guide leads to a well ordered QUE:44BP:22TP stoichiometric ternary solid (Fig. 7 ▸), which is not a solid solution. Tetrathiofulvalene (TTF) as a template highlights an efficient selection of one of four possible synthons, namely *synthon B* that is associated with *Conf 6B*, from the corresponding libraries in solution (Fig. 8 ▸). Both this conformation and this synthon could be termed virtual in the context of this QUE:44BP landscape because they were not observed in this system until the isolation of this particular solid. We extended this ternary design argument with the QUE:DPE-I cocrystal landscape. This exercise highlights recurring molecular conformations like *Conf 5B*, *Conf 6B* and *Conf 7B* in several crystal forms during landscape exploration. The shape–size mimicry of 22TP with 44BP once again acts as a guide for the selection of *Conf 5B* and *synthon E* in the prenucleation events. These precise selections in supramolecular synthesis facilitate the emergence of a porous molecular arrangement and helps to isolate a QUE:DPE-I:22TP ternary solid. Unlike the QUE:44BP:22TP ternary solid, the ‘longer’ coformer provides a larger porous cavity in the QUE:DPE-I binary cocrystal that can efficiently accommodate a 22TP molecule that has both orientational and positional disorder in the pocket (Fig. 7 ▸). Like the TTF template in QUE:44BP, pyrene (PYR) plays a characteristic role in QUE:DPE-I for the amplification of ‘virtual’ *Conf 6B* and *synthon B* chemical features in the ternary solid (Fig. 8 ▸).

**Figure 9:**
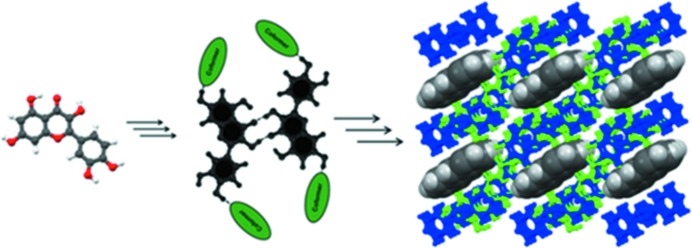
Ternary design strategy. Anthracene (ANT) as a template molecule in QUE:DPE-I cocrystal. This solid shows a unique selection of the globally virtual *Conf 1A* conformation and supramolecular synthon, *synthon J*, in the crystal structure.

We also conceptualized the role of anthracene (ANT) as a template molecule and obtained the respective 2:2:1 ternary solid (Fig. 9 ▸). This crystal structure again highlights the virtuality of conformation and synthon selection from the respective libraries. This conformation amplification in the QUE:DPE-I:ANT ternary solid is, however, different from the previously discussed virtual chemical features because neither the conformation *Conf 1A* or the *synthon J* had been observed by us previously *in any of the quercetin cocrystal landscapes studied here*. Accordingly we term this conformation and this synthon as being *globally virtual*.

## 4. Conclusions   

In conclusion, the isolation of different crystal forms in a quercetin cocrystal system is an illustration of combinatorial crystal synthesis in that there is an inherent selection of a particular molecular conformation and a particular supramolecular synthon in any given case. The principles of combinatorial chemistry may be profitably extended to supramolecular synthesis of solids and the ensuing events may be logically extended to the entire crystallization process prior to nucleation. The absence of concomitant crystallization of different crystal forms in any single experiment shows that the selection is quite efficient. We have recently described the formation of cocrystal polymorphs as combinatorial synthesis based solely on selection from a synthon library. Here we have extended the idea to a selection of both molecular conformation and supramolecular synthon. The convergent nature of the process is further reinforced by the use of template molecules to generate ternary cocrystals, a normally difficult task. Crystallization is known since antiquity as a purification technique which proceeds with efficient exclusion of impurities. The formation of multi-component crystals seems to fly in the face of this reality. However, a closer inspection of the events that take place in the quercetin cocrystal landscapes convey the feeling that the selection of a single conformation and a single synthon is what effectively constitutes the ‘purifying step’ in these crystallizations. Which comes earlier, conformation selection or synthon selection, is hard to say, but formation of a multi-component crystal is then practically inevitable because of enthalpic factors that favour multi-molecular recognition. We feel that these observations open the way to synthesis of ternary and higher component molecular crystals.

## Supplementary Material

Crystal structure: contains datablock(s) QTMP_I, QTMP_II, QTMP_III, QTMP_IV, QBP_I, QBP_II, QBP_III, QBP_IV, QDP_I, QDP_II, QDP_III, QDP_IV, QDP_V, QDPE_I, QDPE_II, QDPE_III, QAP_1, QAP_II, QPH_I, QPH_II, QPH_III, QPH_IV, QPH_V, QBP_22TP, QBP_TTF, QDP_22TP, QDP_PYR, QDP_ANT. DOI: 10.1107/S2052252515009884/lc5063sup1.cif

Supporting figures and tables. DOI: 10.1107/S2052252515009884/lc5063sup2.pdf

CCDC references: 1035751, 1035752, 1035753, 1035754, 1035755, 1035756, 1035757, 1035758, 1035759, 1035760, 1035761, 1035762, 1035763, 1035764, 1035765, 1035766, 1035767, 1035768, 1035769, 1035770, 1035771, 1035772, 1035773, 1035774, 1035775, 1035776, 1035777, 1035778

## Figures and Tables

**Figure 3:**
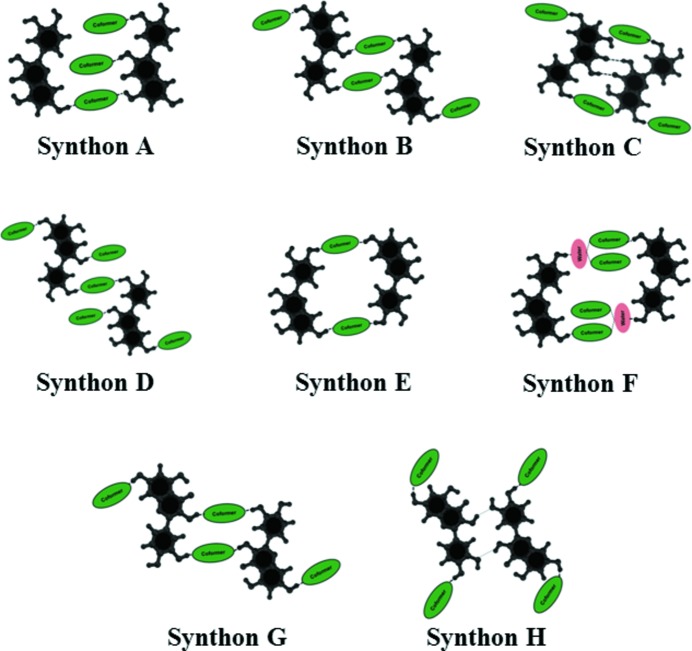
Supramolecular synthons in quercetin cocrystal landscapes. All the members of this synthon library may be interpreted based on O—H⋯O and/or O—H⋯N hydrogen bonding patterns, but each synthon is distinct from any of the others based on topological considerations.

## Footnotes

1 Crystal structures which are neither porous nor helical are considered to be non-porous.
