# Supplementary material for: Combinatorial selection of molecular conformations and supramolecular synthons in quercetin cocrystal landscapes: a route to ternary solids
Source: IUCrJ. 2015 Jun 11;2(Pt 4):402–8. doi: 10.1107/S2052252515009884 (PMC4491312; doi:10.1107/S2052252515009884)
Supplement: Supplementary file 2 [file m-02-00402-sup2.pdf]

IUCrJ

Volume 2 (2015)

Supporting information for article:

Combinatorial selection of molecular conformations and supramolecular synthons in quercetin cocrystal landscapes. A route to ternary solids

Ritesh Dubey and Gautam R. Desiraju

Combinatorial selection of molecular conformations and supramolecular synthons in quercetin cocrystal landscapes. A route to ternary solids

Ritesh Dubey and Gautam R. Desiraju*
Solid State and Structural Chemistry Unit,
Indianstitute of Science, Bangalore 560 012, India

1. Virtual library of molecular conformations
2. Coformers used in the studies
3. Experimental details
4. Normalized hydrogen bond distances
5. Crystallographic tables of the experimental structures

S1. Virtual library of molecular conformations--- M062x/6-31++g(d,p) level calculation

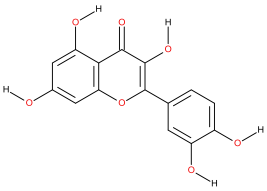<br>Conf 1A<br>0.84 kcal/mol	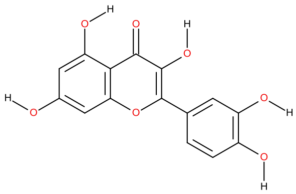<br>Conf 1B<br>1.09 kcal/mol	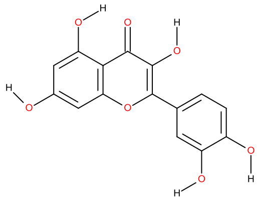<br>Conf 2A<br>0.63 kcal/mol	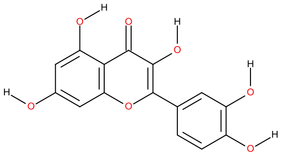<br>Conf 2B<br>0.00 kcal/mol
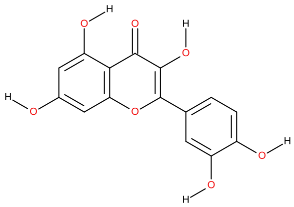<br>Conf 3A<br>4.98 kcal/mol	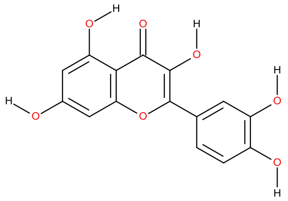<br>Conf 3B<br>4.63 kcal/mol	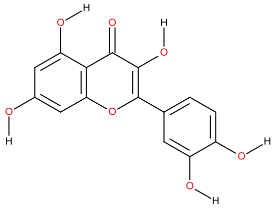<br>Conf 5A<br>1.08 kcal/mol	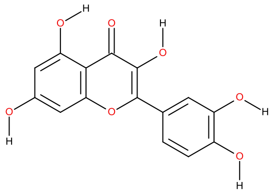<br>Conf 5B<br>1.55 kcal/mol
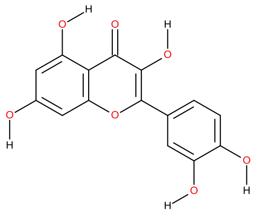	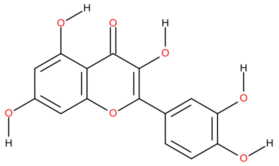	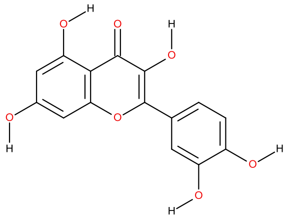	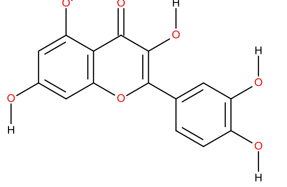

Conf 6A	Conf 6B	Conf 7A	Conf 7B
1.27 kcal/mol	0.36 kcal/mol	5.51 kcal/mol	5.09 kcal/mol

S2. Coformers used in the study

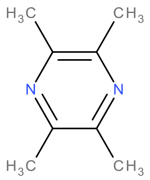

TMP

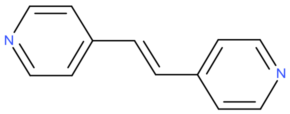

DPE-I

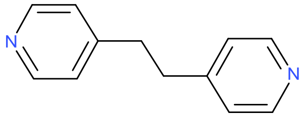

DPE-II

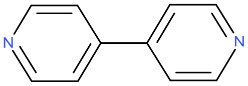

44BP

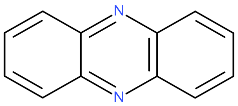

PHE

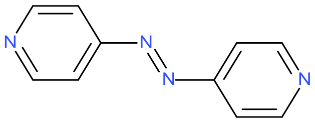

44AP

S3. Experimental details

a) *QUE:TMP (Form I)*: Yellow plate shaped crystals were grown from (1:1) stoichiometric ratios of the compounds using DMSO as a solvent after fifteen days.

b) *QUE:TMP (Form II)*: Yellow plate shaped crystals were grown from (2:1) stoichiometric ratios of the compounds using DMF as a solvent after ten days.

c) *QUE:TMP (Form III)*: Yellow thin plate shaped crystals were grown from (3:1) stoichiometric ratios of the compounds using 1,4-dioxane as a solvent after six days.

d) *QUE:TMP (Form IV)*: Yellow thin plate shaped crystals were grown from (3:1) stoichiometric ratios of the compounds using THF solvent after three days.

e) *QUE:44BP (Form I)*: Yellow thin plate shaped crystals were obtained from (1:1) stoichiometric ratios of the compounds using 1,4-dioxane as solvent after five-six days.

f) *QUE:44BP (Form II)*:Yellow thin plate shaped crystals were grown from (1:1) stoichiometric ratios of the compounds using THF solvent after three days.

g) *QUE:44BP (Form III)*: Yellow plate shaped crystals were grown from (1:1) stoichiometric ratios of the compounds using DMF solvent after ten days.

h) *QUE:44BP (Form IV)*: Monohydrate yellow plate shaped crystals were grown from (1:4) stoichiometric ratios of the compounds using DMSO solvent after ten to fifteen days.

i) *QUE:DPE-I (Form I)*: Yellow plate shaped crystals were grown from (1:1) stoichiometric ratios of the compounds using mixture of 1,4-dioxane-MeOH solvents after six days.

j) *QUE:DPE-I (Form II)*: Yellow plate shaped crystals were grown from (1:1) stoichiometric ratios of the compounds using THF solvent after three days.

k) *QUE:DPE-I (Form III)*:Yellow plate shaped crystals were grown from (2:1) stoichiometric ratios of the compounds using DMF solvent after six days.

l) *QUE:DPE-I (Form IV)*: Yellow plate shaped crystals were grown from (1:1) stoichiometric ratios of the compounds using DMSO solvent, after twelve to fifteen days.

m) *QUE:DPE-I (Form V)*: We utilized the liquid diffusion technique where, the (1:1) solution of QUE:DPE-I was layered with toluene solution of biphenyl. Yellow-red plate shaped diffraction quality crystals were obtained after ten to twelve days.

n) *QUE:DPE-II (Form I)*: Yellow plate shaped crystals were grown from (3:2) stoichiometric ratios of the compounds using mixture of THF-MeOH solvents after four days.

o) *QUE:DPE-II (Form II)*: Yellow plate shaped crystals were grown from (1:1) stoichiometric ratios of the compounds using DMF solvent after ten days.

p) *QUE:DPE-II (Form III)*: Yellow plate shaped crystals were grown from (2:1) stoichiometric ratios of the compounds using DMF solvent after ten to twelve days.

q) *QUE:44AP (Form I)*: Red plate shaped crystals were grown from (2:1) stoichiometric ratios of the compounds using THF solvent after four days.

r) *QUE:44AP (Form II)*: Red plate shaped crystals were grown from (2:1) stoichiometric ratios of the compounds using DMF solvent after ten days.

s) *QUE:PHE (Form I)*: Red plate shaped crystals were grown from (1:1:1) QUE:PHE:ANT stoichiometric ratios using 1,4-dioxane solvents after seven days.

t) *QUE:PHE (Form II)*: Yellow-red plate shaped crystals were grown from (1:1) stoichiometric ratios of the compounds using mixture of CH_3CN:MeOH solvents after four days.

u) *QUE:PHE (Form III)*: Red plate shaped crystals were grown from (1:2) stoichiometric ratios of the compounds using MeOH solvent after four to five days.

v) *QUE:PHE (Form IV)*: Red plate shaped crystals were grown from (1:1) QUE:PHE stoichiometric ratios using mixture of CH_3CN:MeOH solvents after ten to twelve days.

w) *QUE:PHE (Form V)*: Yellow-red plate shaped crystals were grown from (1:1:1) QUE:PHE:22TP stoichiometric ratios using CH_3CN solvent in cold conditions (5°C) after twelve days.

x) *QUE:44BP:22TP*: We utilized the liquid diffusion technique where, the (1:1) *i*-PrOH solution of QUE:44BP was layered with toluene solution of 22TP. Yellow-red plate shaped diffraction quality crystals were obtained after twelve to fifteen days.

y) *QUE:44BP:TTF*: We utilized the liquid diffusion technique where, the (1:1) EtOH solution of QUE:44BP was layered with toluene solution of TTF. Yellow-red plate shaped diffraction quality crystals were obtained after twelve to fifteen days.

z) *QUE:DPE-I:22TP*: We utilized the liquid diffusion technique where, the (1:1) *i*-PrOH solution of QUE:DPE-I was layered with toluene solution of 22TP. Yellow plate shaped diffraction quality crystals were obtained after twelve to fifteen days.

aa) *QUE:DPE-I:PYR:* We utilized the liquid diffusion technique where, the (1:1) *i*-PrOH solution of QUE:DPE-I was layered with toluene solution of PYR. Yellow plate shaped diffraction quality crystals were obtained after twelve to fifteen days.

bb) *QUE:DPE-I:ANT:* We utilized the liquid diffusion technique where, the (1:1) *i*-PrOH solution of QUE:DPE-I was layered with toluene solution of ANT. Yellow plate shaped diffraction quality crystals were obtained after twelve to fifteen days.

	Stoichiometric Ratios	Solvent	Molecular Conformation	Supramolecular Synthon
QUE:TMP (Form I)	1:1	DMSO	Conf 6B	Synthon A
QUE:TMP (Form II)	2:1	DMF	Conf 6B	Synthon B
QUE:TMP (Form III)	3:1	1,4-Dioxane	Conf 5B	Synthon E

QUE:TMP (Form IV)	3:1	THF	Conf 5B	Synthon E
QUE:44BP (Form I)	1:1	1,4-Dioxane	Conf 5B	Synthon E
QUE:44BP (Form II)	1:1	THF	Conf 5B	Synthon E
QUE:44BP (Form III)	1:1	DMF	Conf 5B	Synthon E
QUE:44BP (Form IV)	1:4	DMSO	Conf 7B	Synthon F
QUE:DPE-I (Form I)	1:1	1,4-Dioxane/MeOH	Conf 5B	Synthon E
QUE:DPE-I (Form II)	1:1	THF	Conf 5B	Synthon E
QUE:DPE-I (Form III)	2:1	DMF	Conf 5B	Synthon E
QUE:DPE-I (Form IV)	1:1	DMSO	Conf 7B	Synthon G
QUE:DPE-I (Form V)	1:1, liquid diffusion with toluene solution of biphenyl	*i*-PrOH	Conf 1B	Synthon H
QUE:DPE-II (Form I)	3:2	THF/MeOH	Conf 5B	Synthon E
QUE:DPE-II (Form II)	1:1	DMF	Conf 7B	Synthon G
QUE:DPE-II (Form III)	2:1	DMF	Conf 6B	Synthon C
QUE:44AP (Form I)	2:1	THF	Conf 5B	Synthon E
QUE:44AP (Form II)	2:1	DMF	Conf 1B	Synthon H
QUE:PHE (Form I)	1:1:1 with ANT	1,4-Dioxane	Conf 5B	Synthon E
QUE:PHE (Form II)	1:1	CH_3CN/MeOH	Conf 5B	Synthon E
QUE:PHE (Form III)	1:2	MeOH	Conf 6B	Synthon D
QUE:PHE (Form IV)	1:1	CH_3CN/MeOH	Conf 7B	Synthon G
QUE:PHE (Form V)	1:1:1 with 22TP	CH_3CN (5°C)	Conf 2A	Synthon I
QUE:44BP:22TP	1:1, liquid diffusion with toluene solution of 22TP	*i*-PrOH	Conf 5B	Synthon E
QUE:44BP:TTF	1:1, liquid diffusion with toluene solution of TTF	EtOH	Conf 6B	Synthon B
QUE:DPE-I:22TP	1:1, liquid diffusion with toluene solution of 22TP	*i*-PrOH	Conf 5B	Synthon E
QUE:DPE-I:ANT	1:1, liquid diffusion with toluene solution of ANT	*i*-PrOH	Conf 1A	Synthon J
QUE:DPE-I:PYR	1:1, liquid diffusion with toluene solution of PYR	*i*-PrOH	Conf 6B	Synthon B

Table S1 Normalized hydrogen bond distances

Compound	D–H···A[#]	r(D–H)/Å	r(D–A)/Å	r(H···A)/Å	∠ D–H···A/º	Symmetry
QUE:TMP	O(1)–H(1O)···N(6)	0.98	2.636(5)	1.67	168	x,-1-y,1/2+z

Form I	O(2)–H(2O)···N(5)	0.98	2.927(5)	2.32	119'	1+x,-y,1/2+z
	O(4)–H(4O)···N(1)	0.98	2.811(4)	1.88	157'	1+x,1+y,z
	O(5)–H(5O)···N(3)	0.98	2.848(5)	1.87	174	1+x,1+y,z
	O(6)–H(60)···O(2)	0.98	2.732(4)	1.79	158'	x,-y,-1/2+z
	C(21)–H(21A)···O(1)	1.08	3.373(5)	2.50	137	x,-1-y,-1/2+z
QUE:TMP Form II	O(1)–H(1O)···N(4)	0.98	2.692(3)	1.71	174	x,-1+y,z
	O(2)–H(2O)···O(5)	0.98	3.068(3)	2.45	121'	x,1/2-y,-1/2+z
	O(4)–H(4O)···N(2)	0.98	2.713(3)	1.75	164'	2-x,1-y,-z
	O(5)–H(5O)···N(1)	0.98	2.810(3)	1.83	175	-
	O(6)–H(60)···O(3)	0.98	2.787(3)	1.86	156'	x,1/2-y,1/2+z
	C(21)–H(21A)···O(2)	1.08	3.318(4)	2.38	144	x,1/2-y,1/2+z
QUE:TMP Form III	O(1)–H(1O)···N(2)	0.98	2.691(2)	1.71	174	-
	O(4)–H(4O)···O(3)	0.98	2.6989(17)	1.82	147'	1-x,-y,-z
	O(5)–H(5O)···O(1)	0.98	2.7593(17)	1.83	157	x,y,-1+z
	O(6)–H(6O)···N(1)	0.98	2.7723(19)	1.79	175	-1-x,1-y,-z
	C(21)–H(21A)···O(2)	1.08	3.386(2)	2.49	140	-1+x,1+y,z
	C(24)–H(24A)···O(9)	1.08	3.546(4)	2.46	178	1-x,1-y,-z
QUE:TMP Form IV	O(1)–H(1O)···N(2)	0.98	2.688(3)	1.71	172	2-x,-y,1-z
	O(4)–H(4O)···O(3)	0.98	2.677(2)	1.76	153'	-x,1-y,1-z
	O(5)–H(5O)···O(1)	0.98	2.766(2)	1.87	149	x,y,1+z
	O(6)–H(6O)···N(1)	0.98	2.765(3)	1.80	166	-
	C(22)–H(22A)···O(2)	1.08	3.404(3)	2.49	142	1-x,1-y,1-z
	C(24)–H(24B)···O(2)	1.08	3.429(4)	2.47	146	1-x,-y,1-z
QUE:44BP Form I	O(1)–H(1O)···N(1)	0.98	2.6635(15)	1.69	172	-x,1-y,-z
	O(4)–H(4O)···O(3)	0.98	2.6750(14)	1.81	145'	1-x,1-y,-z
	O(6)–H(5O)···O(1)	0.98	2.7455(12)	1.82	156	x,y,1+z
	O(7)–H(6O)···N(2)	0.98	2.6837(15)	1.70	174	-1+x,1+y,z
	C(22)–H(22)···O(6)	1.08	3.2685(16)	2.37	139	1-x,1-y,1-z
QUE:44BP Form II	O(1)–H(1O)···N(1)	0.98	2.647(4)	1.67	173	-1+x,y,z
	O(4)–H(4O)···O(3)	0.98	2.678(3)	1.80	147'	1-x,1-y,1-z
	O(5)–H(5O)···O(1)	0.98	2.730(3)	1.83	151	x,y,1+z
	O(6)–H(6O)···N(2)	0.98	2.697(4)	1.72	173	-x,2-y,1-z
	C(25)–H(25)···O(5)	1.08	3.250(4)	2.34	141	x,y,-1+z
QUE:44BP Form III	O(1)–H(1O)···N(1)	0.98	2.663(2)	1.68	175	-1-x,2-y,1-z
	O(4)–H(4O)···O(3)	0.98	2.678(2)	1.80	147'	1-x,1-y,1-z
	O(5)–H(5O)···O(1)	0.98	2.753(2)	1.82	157	x,y,1+z
	O(6)–H(6O)···N(2)	0.98	2.719(2)	1.74	174	-
	C(17)–H(17)···O(5)	1.08	3.300(3)	2.36	144	-x,2-y,2-z
QUE:44BP Form IV	O(1)–H(1O)···N(5)	0.98	2.6631(19)	1.68	176	1-x,1-y,1-z
	O(4)–H(4O)···N(3)	0.98	2.7005(17)	1.78	155'	-
	O(5)–H(5O)···N(4)	0.98	2.7533(18)	1.77	175	-1-x,2-y,-z

	O(6)–H(6O)···O(8)	0.98	2.6567(17)	1.68	173	-
	O(8)–H(8A)···N(6)	0.98	2.832(2)	1.89	160	1+x,y,z
	O(8)–H(8B)···N(1)	0.98	2.896(2)	1.95	160	-
	C(40)–H(40)···O(3)	1.08	3.271(2)	2.42	134	1+x,-1+y,z
QUE:DPE-I Form I	O(1)–H(1O)···N(1)	0.98	2.655(3)	1.67	175	-x,1-y,-z
	O(4)–H(4O)···O(3)	0.98	2.759(2)	1.89	146'	1-x,-y,-z
	O(5)–H(5O)···O(1)	0.98	2.7624(19)	1.84	156	x,y,1+z
	O(6)–H(6O)···N(2)	0.98	2.694(3)	1.71	175	-
	C(14)–H(14)···O(8)	1.08	3.416(3)	2.48	144	-1+x,y,z
	C(26)–H(26)···O(2)	1.08	3.350(2)	2.40	145	-x,1-y,-z
	C(31)–H(31A)···O(7)	1.08	3.474(3)	2.51	147	x,1+y,1+z
QUE:DPE-I Form II	O(1)–H(1O)···N(2)	0.98	2.657(3)	1.68	173	-
	O(4)–H(4O)···O(3)	0.98	2.752(2)	1.91	142'	-x,1-y,1-z
	O(5)–H(5O)···O(1)	0.98	2.746(2)	1.85	150	x,y,1+z
	O(6)–H(6O)···N(1)	0.98	2.704(3)	1.73	171	3-x,-y,1-z
	C(14)–H(14)···O(8)	1.08	3.383(4)	2.48	140	2-x,1-y,1-z
	C(20)–H(20)···O(2)	1.08	3.375(3)	2.42	146	1-x,1-y,-z
QUE:DPE-I Form III	O(1)–H(1O)···N(1)	0.98	2.642(3)	1.66	176	-
	O(4)–H(4O)···O(3)	0.98	2.758(3)	1.91	143'	2-x,-y,-z
	O(5)–H(5O)···O(1)	0.98	2.769(3)	1.88	149	x,y,-1+z
	O(6)–H(6O)···N(2)	0.98	2.693(3)	1.71	178	-1-x,1-y,-z
	C(16)–H(16)···O(8)	1.08	3.203(5)	2.20	154	1-x,1-y,-z
	C(20)–H(20)···O(2)	1.08	3.325(3)	2.35	149	1-x,-y,1-z
QUE:DPE-I Form IV	O(1)–H(1O)···N(2)	0.98	2.7238(19)	1.75	171	-x,1-y,1-z
	O(4)–H(4O)···N(4)	0.98	2.6921(19)	1.74	161'	1-x,1-y,-z
	O(5)–H(5O)···N(3)	0.98	2.7283(19)	1.75	172	x,1+y,z
	O(6)–H(6O)···N(1)	0.98	2.759(2)	1.78	173	x,3/2-y,1/2+z
	C(28)–H(28)···O(5)	1.08	3.3389(19)	2.41	143	1-x,1-y,1-z
	C(37)–H(37)···O(3)	1.08	3.014(2)	2.34	119	1-x,1-y,-z
QUE:DPE-I Form V	O(1)–H(1O)···N(1)	0.98	2.763(3)	1.78	173	1/2-x,1/2+y,1/2-z
	O(4)–H(4O)···O(5)	0.98	2.690(2)	1.79	151'	-x,y,-1/2-z
	O(5)–H(5O)···O(2)	0.98	2.797(2)	1.94	144'	-1/2+x,3/2-y,-1/2+z
	O(6)–H(6O)···N(2)	0.98	2.663(3)	1.71	162	-1/2-x,1/2-y,-z
	C(4)–H(4)···N(1)	1.08	3.287(3)	2.50	128	1/2-x,1/2+y,1/2-z
	C(20)–H(20)···O(3)	1.08	3.294(3)	2.50	130	1/2-x,1/2-y,-z
QUE:DPE-II Form I	O(1)–H(1O)···N(2)	0.98	2.657(2)	1.68	172	-x,1-y,2-z
	O(4)–H(4O)···O(3)	0.98	2.722(2)	1.88	141'	2-x,-y,2-z
	O(6)–H(5O)···O(1)	0.98	2.7887(19)	1.89	151	x,y,-1+z
	O(7)–H(6O)···N(1)	0.98	2.702(2)	1.72	178	-1+x,y,z
	C(17)–H(17)···O(1)	1.08	3.320(3)	2.47	134	1-x,1-y,2-z

	C(25)–H(25)···O(2)	1.08	3.426(2)	2.49	144	-1+x,1+y,-1+z
QUE:DPE-II Form II	O(1)–H(1O)···N(2)	0.98	2.6962(18)	1.72	174	1-x,1-y,-z
	O(4)–H(4O)···N(3)	0.98	2.6367(18)	1.68	163'	-
	O(5)–H(5O)···N(4)	0.98	2.7009(18)	1.73	170	-x,1-y,1-z
	O(6)–H(6O)···N(1)	0.98	2.7236(18)	1.75	171	x,1/2-y,-1/2+z
	C(4)–H(4)···O(2)	1.08	3.4732(19)	2.43	160	1-x,2-y,1-z
	C(28)–H(28)···O(6)	1.08	3.295(2)	2.43	136	x,1/2-y,1/2+z
	C(37)–H(37)···O(5)	1.08	3.386(2)	2.30	177	x,y,1+z
	C(39)–H(39)···O(6)	1.08	3.253(2)	2.41	134	-x,1/2+y,1/2-z
QUE:DPE-II Form III	O(1)–H(1O)···N(3)	0.98	2.666(3)	1.70	166	1-x,-1/2+y,5/2-z
	O(4)–H(4O)···O(10)	0.98	2.7504(19)	1.90	143'	-
	O(5)–H(5O)···N(4)	0.98	2.742(3)	1.76	173	-x,1-y,2-z
	O(6)–H(6O)···O(4)	0.98	2.883(2)	2.14	131'	-x,1/2+y,5/2-z
	O(8)–H(8O)···N(1)	0.98	2.619(2)	1.65	167	-x,-y,2-z
	O(9)–H(9O)···O(6)	0.98	3.033(2)	2.41	120'	-x,-1/2+y,5/2-z
	O(11)–H(11O)···O(3)	0.98	2.7228(19)	1.82	150	-
	O(12)–H(12O)···N(2)	0.98	2.699(2)	1.72	170	1-x,1/2+y,5/2-z
	O(13)–H(13O)···O(8)	0.98	2.9042(19)	2.09	139	1+x,y,z
	C(4)–H(4)···O(7)	1.08	3.380(2)	2.43	146	1-x,-1/2+y,5/2-z
	C(36)–H(36B)···O(14)	1.08	3.461(3)	2.45	154	-
	C(47)–H(47)···O(13)	1.08	3.397(3)	2.36	159	1-x,-y,2-z
	C(50)–H(50)···O(11)	1.08	3.460(3)	2.43	158	1-x,-1/2+y,5/2-z
	C(54)–H(54)···O(9)	1.08	3.232(3)	2.32	141	-x,-1/2+y,5/2-z
QUE:44AP Form I	O(1)–H(1O)···N(4)	0.98	2.685(6)	1.70	175	1-x,1-y,-z
	O(4)–H(4O)···O(3)	0.98	2.729(5)	1.80	157'	2-x,-y,-z
	O(5)–H(5O)···O(1)	0.98	2.756(4)	1.85	151	x,y,1+z
	O(6)–H(6O)···N(1)	0.98	2.719(6)	1.74	175	-1+x,y,z
	C(14)–H(14)···O(8)	1.08	3.267(7)	2.39	137	1-x,1-y,-z
	C(21)–H(21)···O(2)	1.08	3.404(5)	2.44	148	x,1+y,1+z
QUE:44AP Form II	O(1)–H(1O)···N(4)	0.98	2.791(3)	1.81	174	-
	O(4)–H(4O)···O(5)	0.98	2.669(2)	1.79	148'	-x,y,-1/2-z
	O(5)–H(5O)···O(2)	0.98	2.759(2)	1.92	141'	-1/2+x,1/2-y,-1/2+z
	O(6)–H(6O)···N(1)	0.98	2.690(3)	1.76	156	-1+x,1-y,-1/2+z
	C(4)–H(4)···N(4)	1.08	3.292(3)	2.50	124	-
	C(24)–H(24)···O(3)	1.08	3.294(3)	2.51	128	x,1-y,1/2+z
QUE:PHE Form I	O(1)–H(1O)···N(2)	0.98	2.760(3)	1.78	173	-x,1-y,1-z
	O(4)–H(4O)···O(8)	0.98	2.704(2)	1.91	136'	-
	O(5)–H(5)···O(1)	0.98	2.765(2)	1.85	153	x,y,-1+z
	O(6)–H(6O)···N(1)	0.98	2.796(3)	1.83	167	-1+x,y,z
	C(17)–H(17)···O(2)	1.08	3.313(3)	2.46	135	1-x,-y,1-z

	C(24)–H(24)⋯O(5)	1.08	3.362(3)	2.45	141	1-x,1-y,-z
QUE:PHE Form II	O(1)–H(1O)⋯N(1)	0.98	2.722(5)	1.74	177	1-x,1-y,1-z
	O(5)–H(5O)⋯O(1)	0.98	2.737(4)	1.80	159	x,y,1+z
	O(6)–H(6A)⋯N(2)	0.98	2.786(5)	1.82	165	1+x,-1+y,1+z
	C(20)–H(20)⋯O(2)	1.08	3.263(6)	2.46	130	-x,2-y,1-z
QUE:PHE Form III	O(1)–H(1O)⋯N(4)	0.98	2.7694(18)	1.81	164	1-x,1-y,1-z
	O(4)–H(4O)⋯N(5)	0.98	2.6997(17)	1.80	150'	-
	O(5)–H(5O)⋯N(7)	0.98	2.9068(17)	1.93	174	-
	O(6)–H(6O)⋯O(8)	0.98	2.6122(18)	1.67	159'	-
	O(8)–H(8O)⋯N(6)	0.98	2.811(2)	1.83	177	-x,2-y,-z
	C(14)–H(14)⋯N(1)	1.08	3.464(2)	2.41	164	-
	C(20)–H(20)⋯O(3)	1.08	3.3386(19)	2.34	152	1+x,-1+y,z
QUE:PHE Form IV	O(1)–H(1O)⋯N(5)	0.98	2.736(2)	1.76	169	-
	O(4)–H(4O)⋯N(11)	0.98	2.850(2)	1.95	152'	3-x,1-y,1-z
	O(5)–H(5O)⋯N(12)	0.98	2.861(2)	1.89	171	x,1+y,1+z
	O(6)–H(6O)⋯O(8)	0.98	2.715(2)	1.74	174	-
	C(19)–H(19)⋯O(6)	1.08	3.347(3)	2.45	139	1+x,-1+y,-1+z
	C(28)–H(28)⋯O(3)	1.08	3.134(2)	2.16	149	-1+x,y,-1+z
QUE:PHE Form V	O(1)–H(1O)⋯N(2)	0.98	2.792(3)	1.85	159	-x,-y,1-z
	O(4)–H(4O)⋯O(3)	0.98	2.699(3)	1.82	147'	2-x,1-y,1-z
	O(5)–H(5O)⋯O(6)	0.98	2.792(3)	1.98	139'	-x,-y,-z
	O(6)–H(6O)⋯N(1)	0.98	2.743(3)	1.78	167	-
	C(18)–H(18)⋯O(7)	1.08	3.458(3)	2.44	156	-1+x,y,z
	C(24)–H(24)⋯N(3)	1.08	3.353(4)	2.33	157	x,-1+y,z
QUE:44BP:22TP	O(1)–H(1O)⋯N(2)	0.98	2.658(3)	1.68	176	2-x,1-y,-z
	O(4)–H(4O)⋯O(3)	0.98	2.667(3)	1.80	145'	-x,2-y,-z
	O(5)–H(5O)⋯O(1)	0.98	2.737(2)	1.80	158	x,y,-1+z
	O(6)–H(6O)⋯N(1)	0.98	2.706(3)	1.73	171	-
	C(17)–H(17)⋯O(5)	1.08	3.275(2)	2.33	144	1-x,1-y,-1-z
QUE:44BP:TTF	O(1)–H(1O)⋯N(1)	0.98	2.672(4)	1.71	165	1-x,-y,1-z
	O(4)–H(4O)⋯N(5)	0.98	2.679(4)	1.73	161'	-
	O(5)–H(5O)⋯N(4)	0.98	2.733(5)	1.75	175	-1+x,y,1+z
	O(6)–H(6O)⋯O(1)	0.98	2.794(3)	1.99	137	x,1+y,z
	O(8)–H(8O)⋯N(2)	0.98	2.693(4)	1.75	159	-x,1-y,1-z
	O(11)–H(11O)⋯N(3)	0.98	2.841(4)	1.91	157'	-
	O(12)–H(12O)⋯N(6)	0.98	2.703(4)	1.72	175	1+x,y,-1+z
	O(13)–H(13O)⋯O(8)	0.98	2.807(3)	1.99	139	x,-1+y,z
	C(4)–H(4)⋯O(5)	1.08	3.342(5)	2.35	152	x,-1+y,z
	C(14)–H(14)⋯S(2)	1.08	3.557(3)	2.64	142	-
	C(15)–H(15)⋯S(3)	1.08	3.439(3)	2.66	128	1-x,-y,1-z

	C(19)–H(19)···O(12)	1.08	3.367(3)	2.36	155	x,1+y,z
	C(41)–H(41)···O(10)	1.08	3.102(4)	2.40	121	-
	C(41)–H(41)···S(6)	1.08	3.625(4)	2.77	136'	1-x,1-y,-z
	C(48)–H(48)···O(2)	1.08	3.051(5)	2.46	113	1+x,1+y,-1+z
QUE:DPE-I:22TP	O(1)–H(1O)···N(1)	0.98	2.659(3)	1.68	175	-
	O(4)–H(4O)···O(3)	0.98	2.730(3)	1.84	148'	-x,1-y,1-z
	O(5)–H(5O)···O(1)	0.98	2.759(3)	1.84	154	x,y,1+z
	O(6)–H(6O)···N(2)	0.98	2.706(4)	1.73	169	3-x,-y,1-z
	C(20)–H(20)···O(2)	1.08	3.344(3)	2.36	150	1-x,1-y,-z
QUE:DPE-I:PYR	O(1)–H(1O)···N(1)	0.98	2.640(4)	1.68	166	-
	O(4)–H(4O)···N(6)	0.98	2.750(4)	1.79	166'	2-x,1-y,-z
	O(5)–H(5O)···N(4)	0.98	2.759(4)	1.79	169	2-x,-y,-z
	O(6)–H(6O)···O(1)	0.98	2.766(4)	1.95	139	x,-1+y,z
	O(8)–H(8O)···N(2)	0.98	2.653(4)	1.69	167	-x,1-y,1-z
	O(11)–H(11O)···N(3)	0.98	2.830(5)	1.99	141'	-
	O(12)–H(12O)···N(5)	0.98	2.732(4)	1.76	170	x,-1+y,z
	O(13)–H(13O)···O(8)	0.98	2.772(4)	1.97	137	x,-1+y,z
	C(4)–H(4)···O(5)	1.08	3.450(5)	2.46	151	x,1+y,z
	C(35)–H(35)···O(6)	1.08	3.449(5)	2.47	150	x,1+y,z
	C(41)–H(41)···O(13)	1.08	3.391(5)	2.41	149	-x,-y,1-z
	C(47)–H(47)···O(10)	1.08	3.093(5)	2.32	127	-
	C(53)–H(53)···O(2)	1.08	3.299(5)	2.35	145	2-x,1-y,-z
	C(55)–H(55)···O(9)	1.08	3.328(5)	2.35	150	-
	C(73)–H(73)···O(3)	1.08	3.313(5)	2.46	135	-1+x,y,z
	C(75)–H(75)···O(1)	1.08	3.506(5)	2.44	168	-
	C(83)–H(83)···O(12)	1.08	3.404(5)	2.46	144	x,1+y,z
QUE:DPE-I:ANT	O(1)–H(1O)···N(2)	0.98	2.692(2)	1.72	168	x,-1+y,1+z
	O(4)–H(4O)···O(3)	0.98	2.731(2)	1.88	143'	x,1-y,2-z
	O(5)–H(5O)···N(1)	0.98	2.692(2)	1.71	172	-
	O(6)–H(6O)···O(1)	0.98	2.872(2)	2.18	126'	1-x,-y,1-z
	C(2)–H(2)···O(6)	1.08	3.138(3)	2.28	135	1-x,-y,1-z
	C(31)–H(31)···O(4)	1.08	3.471(3)	2.48	152	-

Table S2 Crystallographic tables for the experimental structures

**Compound**	**QUE:TMP Form I**	**QUE:TMP Form II**	**QUE:TMP Form II**	**QUE:TMP Form IV**	**QUE:44BP Form I**	**QUE:44BP Form II**	**QUE:44BP Form III**
**CCDC No.**	1035762	1035763	1035765	1035764	1035766	1035767	1035768
**Molecular formula**	$C_{39}H_{46}N_6O_7$	$C_{31}H_{34}N_4O_7$	$C_{27}H_{30}N_2O_9$	$C_{27}H_{30}N_2O_8$	$C_{29}H_{26}N_2O_9$	$C_{29}H_{26}N_2O_8$	$C_{26}H_{22}N_2O_8$
**Formula weight**	710.82	574.62	526.53	510.53	546.52	530.52	536.51
**Crystal system**	Monoclinic	Monoclinic	Triclinic	Triclinic	Triclinic	Triclinic	Triclinic
**Space group**	*P*c	$P2_1$/c	*P*1	*P*1	*P*1	*P*1	*P*1
***a* (Å)**	9.0760(13)	15.831(2)	9.3506(7)	9.3202(10)	9.8334(7)	9.806(2)	9.844(5)
***b* (Å)**	8.9616(13)	9.3122(13)	10.9120(8)	10.7538(12)	10.7098(8)	10.778(2)	10.636(6)
***c* (Å)**	22.501(3)	20.211(3)	13.002(1)	12.9869(14)	13.0790(9)	13.055(3)	13.071(7)
**α (°)**	90	90	76.798(5)	79.471(6)	102.815(7)	102.833(7)	98.324(7)
**β (°)**	93.554(5)	104.415(7)	84.048(6)	85.133(6)	95.031(7)	94.525(7)	94.601(7)
**γ (°)**	90	90	81.580(6)	82.377(6)	106.846(7)	106.943(7)	107.947(8)
**V (Å^3)**	1826.6(4)	2885.7(7)	1274.26(17)	1266.0(2)	1268.30(17)	1271.6(5)	1276.9(12)
**ρcalc (g/cm^3)**	1.292	1.323	1.372	1.339	1.431	1.365	1.395
***F*(000)**	756	1216	556	540	572	540	558
**μ (mm^{-1})**	0.090	0.095	0.104	0.099	0.107	0.101	0.101
***T* (K)**	150K	150K	150K	150K	150K	150K	150K
**λ (Å)**	0.71073	0.71073	0.71073	0.71073	0.71073	0.71073	0.71073
**Reflns. collected**	18413	22578	13650	13412	13454	10628	13246
**Unique reflns.**	8351	6558	5832	5779	5791	4943	5767
**Completeness (%)**	99.2	99.2	99.9	99.7	99.7	99.3	99.0
**R_{int}**	0.062	0.077	0.034	0.030	0.016	0.037	0.022
**R_1 (F^2)**	0.0579	0.0628	0.0523	0.0698	0.0400	0.0748	0.0427
**wR_2(F^2)**	0.1304	0.1626	0.1587	0.2016	0.1097	0.2137	0.1137
**Goodness-of-fit**	1.04	0.96	0.93	1.05	1.05	1.06	1.04
**Resolution (2θ)**	54	54	54	54	54	54	54

Compound	QUE:44BP Form IV	QUE:DPE-I Form I	QUE:DPE-I Form II	QUE:DPE-I Form III	QUE:DPE-I Form IV	QUE:DPE-I Form V	QUE:DPE-II Form I
**CCDC No.**	1035769	1035770	1035771	1035772	1035773	1035774	1035775
**Molecular formula**	$C_{45}H_{36}N_6O_8$	$C_{31}H_{28}N_2O_9$	$C_{31}H_{28}N_2O_8$	$C_{30}H_{26}N_3O_8$	$C_{39}H_{30}N_4O_7$	$C_{27}H_{20}N_2O_7$	$C_{31}H_{30}N_2O_8$
**Formula weight**	788.80	572.55	556.55	556.54	666.67	484.45	558.57
**Crystal system**	Triclinic	Triclinic	Triclinic	Triclinic	Monoclinic	Monoclinic	Triclinic
**Space group**	*P*1	*P*1	*P*1	*P*1	$P2_1$/c	*C*2/c	*P*1
***a* (Å)**	10.0222(12)	10.1899(9)	10.0381(7)	9.9867(15)	18.0656(11)	21.831(5)	9.8782(8)
***b* (Å)**	12.8756(16)	11.6532(11)	11.5707(8)	11.2949(12)	11.6584(6)	12.904(3)	11.5881(10)
***c* (Å)**	15.0597(18)	13.0214(12)	13.0194(9)	13.040(2)	16.6610(1)	15.876(4)	13.041(1)
**α (°)**	81.685(6)	97.351(7)	76.744(5)	79.558(6)	90	90	76.595(5)
**β (°)**	83.118(6)	95.446(7)	84.502(6)	85.192(6)	115.565(2)	101.912(7)	85.792(6)
**γ (°)**	76.273(5)	115.237(8)	65.573(5)	66.082(5)	90	90	67.035(5)
**V (Å^3)**	1860.6(4)	1367.8(2)	1340.13(17)	1322.2(4)	3165.5(3)	4376.1(18)	1336.8(2)
**ρcalc (g/cm^3)**	1.408	1.390	1.379	1.398	1.399	1.471	1.388
***F*(000)**	824	600	584	582	1392	2016	588
**μ (mm^{-1})**	0.099	0.103	0.100	0.103	0.098	0.108	0.101
***T* (K)**	150K	150K	150K	150K	150K	150K	150K
**λ (Å)**	0.71073	0.71073	0.71073	0.71073	0.71073	0.71073	0.71073
**Reflns. collected**	19338	14650	14382	13781	27138	18633	14292
**Unique reflns.**	8502	6248	6106	6040	7197	5006	6112
**Completeness (%)**	99.7	99.7	99.8	99.7	99.1	99.5	99.8
**R_{int}**	0.031	0.035	0.034	0.052	0.038	0.071	0.036
**R_1 (F^2)**	0.0452	0.0511	0.0566	0.0634	0.0468	0.0545	0.0544
**wR_2(F^2)**	0.1343	0.1323	0.1618	0.1776	0.1188	0.1497	0.1479
**Goodness-of-fit**	0.88	1.02	1.03	1.02	1.04	1.05	1.04
**Resolution (2θ)**	54	54	54	54	54	54	54

Compound	QUE:DPE-II	QUE:DPE-II	QUE:44AP	QUE:44AP	QUE:PHE	QUE:PHE	QUE:PHE

	Form II	Form III	Form I	Form II	Form I	Form II	Form III
**CCDC No.**	1035776	1035777	1035778	1035751	1035752	1035753	1035754
**Molecular formula**	$C_{39}H_{34}N_4O_7$	$C_{27}H_{22}N_2O_7$	$C_{29}H_{26}N_4O_8$	$C_{25}H_{18}N_4O_7$	$C_{31}H_{26}N_2O_9$	$C_{33}H_{22}N_3O_7$	$C_{58}H_{42}N_7O_8$
**Formula weight**	670.70	486.47	558.54	486.43	570.54	572.54	964.99
**Crystal system**	Monoclinic	Monoclinic	Triclinic	Monoclinic	Triclinic	Triclinic	Triclinic
**Space group**	$P2_1/c$	$P2_1/c$	$P1$	$C2/c$	$P1$	$P1$	$P1$
***a* (Å)**	20.028(3)	13.3624(4)	10.0918(17)	21.647(3)	8.1859(13)	8.416(3)	9.9945(3)
***b* (Å)**	10.5025(13)	10.5540(4)	11.4244(19)	12.8623(16)	12.787(2)	12.297(4)	13.4346(4)
***c* (Å)**	16.5786(18)	32.5542(10)	13.052(2)	15.712(2)	13.173(2)	13.111(4)	17.8220(5)
α (°)	90	90	98.573(7)	90	83.634(6)	87.100(6)	81.016(1)
β (°)	108.851(4)	95.517(3)	95.190(7)	101.543(7)	83.848(6)	84.103(6)	78.335(1)
γ (°)	90	90	115.078(8)	90	73.246(5)	73.503(5)	86.046(1)
V (Å^3)	3300.2 (7)	4569.8(3)	1327.7(4)	4286.2(10)	1308.1(4)	1293.8(7)	2313.09(12)
$\rho calc$ (g/cm^3)	1.350	1.414	1.397	1.508	1.449	1.470	1.385
F(000)	1408	2032	584	2016	596	594	1006
μ (mm^{-1})	0.094	0.103	0.104	0.113	0.108	0.105	0.094
***T* (K)**	150K	150K	150K	150K	150K	150K	150K
λ (Å)	0.71073	0.71073	0.71073	0.71073	0.71073	0.71073	0.71073
**Reflns. collected**	36152	43116	11363	24298	12319	10572	29578
**Unique reflns.**	7537	10476	5197	4173	5126	5028	10501
**Completeness (%)**	99.4	99.9	99.6	99.0	99.8	98.8	98.6
R_{int}	0.053	0.050	0.074	0.051	0.032	0.128	0.033
R_1 (F^2)	0.0450	0.0530	0.0898	0.0517	0.0544	0.0845	0.0456
wR_2(F^2)	0.1133	0.1499	0.2647	0.1362	0.1435	0.2156	0.1203
**Goodness-of-fit**	1.04	1.02	1.17	0.89	1.08	0.99	1.03
**Resolution (2θ)**	54	54	54	54	54	54	54
**Compound**	**QUE:PHE Form IV**	**QUE:PHE Form V**	**QUE:44BP 22TP**	**QUE:44BP TTF**	**QUE:DPE-I 22TP**	**QUE:DPE-I PYR**	**QUE:DPE-I ANT**
**CCDC No.**	1035755	1035756	1035757	1035758	1035759	1035760	1035761

**Molecular formula**	$C_{33}H_{24}N_3O_8$	$C_{33}H_{22}N_3O_7$	$C_{29}H_{21}N_2O_7S$	$C_{69}H_{50}N_6O_{14}S_6$	$C_{31}H_{23}N_2O_7S$	$C_{90}H_{63}N_6O_{14}$	$C_{34}H_{24}N_2O_7$
**Formula weight**	590.56	572.54	541.55	1379.57	567.59	1452.46	572.55
**Crystal system**	Triclinic	Triclinic	Triclinic	Triclinic	Triclinic	Triclinic	Triclinic
**Space group**	*P*1	*P*1	*P*1	*P*1	*P*1	*P*1	*P*1
***a* (Å)**	9.4800(7)	4.9830	9.8066(9)	12.547(2)	9.974	12.664	10.5919(6)
***b* (Å)**	11.2614(9)	15.3810	10.4874(10)	13.299(2)	11.421	13.235	11.6067(6)
***c* (Å)**	13.5129(10)	18.1420	13.0073(12)	18.490(3)	13.009	22.191	11.6919(7)
α (°)	104.785(4)	112.210	97.993(7)	94.246(7)	78.510	104.030	85.133(4)
β (°)	108.855(4)	94.990	94.646(7)	90.204(6)	85.220	96.590	72.938(3)
γ (°)	90.687(4)	94.920	108.150(8)	98.776(7)	66.390	104040	75.632(3)
V (Å^3)	1312.95(18)	1271.766	1247.8(2)	3040.5(8)	1330.627	3440.593	1331.01(13)
$\rho calc$ (g/cm^3)	1.489	1.495	1.441	1.507	1.379	1.402	1.429
F(000)	610	594	562	1428	572	1514	596
μ (mm^{-1})	0.108	0.107	0.184	0.302	0.174	0.096	0.101
***T* (K)**	150K	150K	150K	150K	150K	150K	150K
λ (Å)	0.71073	0.71073	0.71073	0.71073	0.71073	0.71073	0.71073
**Reflns. collected**	14918	13154	13323	31942	14146	35694	20837
**Unique reflns.**	5100	5827	5709	13902	6078	15699	5395
**Completeness (%)**	99.0	99.6	99.8	99.6	99.8	99.5	99.0
R_{int}	0.042	0.066	0.031	0.064	0.060	0.096	0.050
R_1 (F^2)	0.0456	0.0614	0.0526	0.0666	0.0679	0.0804	0.0501
wR_2(F^2)	0.1226	0.1549	0.1421	0.1877	0.2020	0.2131	0.1400
**Goodness-of-fit**	0.99	0.97	1.06	1.03	1.04	1.02	1.04
**Resolution (2θ)**	52	54	54	54	54	54	52
